# Ferulic acid: extraction, estimation, bioactivity and applications for human health and food

**DOI:** 10.1002/jsfa.13931

**Published:** 2024-10-02

**Authors:** Mukul Kumar, Deepika Kaushik, Shubham Shubham, Ashwani Kumar, Vishal Kumar, Emel Oz, Charles Brennan, Maomao Zeng, Charalampos Proestos, Kenan Çadırcı, Muharrem Bayrak, Tahra Elobeid, Sercan Karav, Fatih Oz

**Affiliations:** ^1^ Department of Food Technology and Nutrition Lovely Professional University Phagwara India; ^2^ Department of Biotechnology, Faculty of Applied Sciences and Biotechnology Shoolini University Solan India; ^3^ Department of Innovation Engineering University of Salento Brindisi Italy; ^4^ Institute of Food Technology Bundelkhand University Jhansi India; ^5^ Department of Food Engineering, Agriculture Faculty Ataturk University Erzurum Turkey; ^6^ RMIT University School of Science Melbourne Victoria Australia; ^7^ State Key Laboratory of Food Science and Technology Jiangnan University Wuxi China; ^8^ International Joint Laboratory on Food Safety Jiangnan University Wuxi China; ^9^ Laboratory of Food Chemistry, Department of Chemistry, School of Sciences National and Kapodistrian University of Athens Athens Greece; ^10^ Department of Internal Medicine, Erzurum Regional Training and Research Hospital Health Sciences University Erzurum Turkey; ^11^ Human Nutrition Department, College of Health Sciences, QU Health Qatar University Doha Qatar; ^12^ Çanakkale Onsekiz Mart University Çanakkale Turkey

**Keywords:** ferulic acid, antiapoptotic, antifibrosis, anti‐inflammatory, antioxidant, bioactive component

## Abstract

Ferulic acid ((*E*)‐3‐(4‐hydroxy‐3‐methoxy‐phenyl) prop‐2‐enoic acid) is a derivative of caffeic acid found in most plants. This abundant phenolic compound exhibits significant antioxidant capacity and a broad spectrum of therapeutic effects, including anti‐inflammatory, antimicrobial, anticancer, antidiabetic, cardiovascular and neuroprotective activities. It is absorbed more quickly by the body and stays in the bloodstream for a longer period compared with other phenolic acids. It is widely used in the food (namely whole grains, fruits, vegetables and coffee), pharmaceutical and cosmetics industries. The current review highlights ferulic acid and its pharmacological activities, reported mechanisms of action, food applications (food preservative, food additive, food processing, food supplements and in food packaging in the form of edible films) and role in human health. In the future, the demand for ferulic acid in the food and pharmaceutical industries will increase. © 2024 The Author(s). *Journal of the Science of Food and Agriculture* published by John Wiley & Sons Ltd on behalf of Society of Chemical Industry.

## INTRODUCTION

Ferulic acid (FA), also known as 3‐(4‐hydroxy‐3‐methoxyphenyl)‐2‐propenoic acid, belongs to the phenolic acid family. It is a derivative of caffeic acid majorly found in beverages (coffee and beer).^1^, ^2^ It is extensively distributed throughout the plant kingdom, particularly in the Ranunculaceae and Gramineae umbrella families, including *Angelica*, *Cimicifuga*, *Spargani rhizoma*, *Ligusticum chuanxiong*, reed root, etc.^3^, ^4^ It is a non‐toxic compound and has numerous physiological effects such as anticancer, antidiabetic, anti‐inflammatory, antioxidant and antimicrobial; for these reasons, it is widely utilized in the food, pharmaceutical and cosmetics industries. It also helps to prevent coronary artery disease, decrease cholesterol and boost sperm viability.^5^ FA is mainly absorbed in the small intestine and is eliminated from the human body with the help of urine. It is utilized as a base material in the synthesis of vanillin and preservatives, and as an interlinking agent with edible films and food gels during the manufacturing process. It also works as a constituent in different products such as sports foods and skin protection products. Chemical synthesis and biological transformation can both be used to make FA.^6^ It is a bioactive component found in a variety of foods such as bamboo shoots, bananas, beetroot, broccoli, cabbage, citrus fruits and juices, coffee, eggplant, grain bran, spinach and whole‐grain foods. In Western countries, the main sources of FA are natural extracts such as coffee, herbs and spices.^7^ FA acts as a scavenger of free radicals, as well as an inhibitor of free radical‐generating enzymes and is also responsible for an increase in scavenger enzyme activity (acrolein). The primary skin structures, fibroblasts, keratinocytes, elastin and collagen are all safeguarded by FA. It inhibits melanogenesis, which decreases the healing process of wounds and also enhances angiogenesis. It is used as a photoprotective agent, which slows down the photo‐aging process of the skin and is a lightening ingredient in skincare products. Its application is limited due to its susceptibility to oxidation.^5^ It is thought to be a better antioxidant than other phenolic acids and stays longer in the blood circulation. FA is utilized as an antioxidant to attenuate oxidative stress and prevent cell damage in muscular tissues. It shows enhanced activity in combination with vitamins A, C and E and slows down the aging process. The powerful radical‐scavenging ability of FA helps in skin‐related diseases due to ultraviolet radiation absorption, having anti‐aging, anti‐pigmentation, anti‐wrinkle, skin whitening and photoprotective properties, for example.^8^ Table 1 lists the FA content of different plant‐based products. The present review discusses the various biotechnological and food applications and health benefits of FA in human life.

**Table 1:** Ferulic acid content in plant‐based products

Food	Ferulic acid mg kg^−1^	References
Wheat bran	700.0	3, 5, 21, 27, 29
Tomato	700.0	
Red beet	250.0	
Cucurbit	220.0	
Wheat flour	150.0	
Oatmeal	145.0	
Soybean	120.0	
Spinach	110.0	
Orange	99.0	
Banana	54.0	
Blackcurrant	15.0	
Blackberry	10.0	

In the literature, several studies have focused on the application of nanomaterials in electrochemical sensing to detect particular and significant antioxidants, both natural and synthetic. In particular, to detect FA, reliable methods include high‐performance liquid chromatography (HPLC),^9^ high‐performance capillary electrophoresis^10^ and thin‐layer chromatography.^11^ However, these techniques may not be well suited from many aspects.^12^, ^13^, ^14^ The electrochemical sensor method is an alternative technique, being a convenient and cost‐effective process, with high detection selectivity.^15^ Graphene has been found to be a superior electrode material for electrochemical sensor and biosensor applications.^9^ Graphene is an attractive material, with novel properties such as thermal and chemical stability, superior biocompatibility, high carrier mobility, and electrical conductivity.^10^, ^11^, ^12^, ^13^, ^14^, ^15^, ^16^, ^17^ Graphene film‐based electrode is crucial to further expand the application of graphene in electrochemical sensors. The performance of the sensor can be remarkably improved because of excellent electrical conductivity, strong adsorptive ability and large effective surface area. The modified electrode can exhibit a high electrocatalytic activity and good selectivity for FA.

## EXTRACTION AND ESTIMATION OF FA

### Extraction of FA

The extraction process to optimize FA was carried out in laboratories: three start‐up weights of wheat bran, four concentrations of sodium hydroxide and three hydrolysis temperatures were employed. The wheat bran was treated with 150 mL sodium hydroxide in an Erlenmeyer flask and the mixture was shaken for 4 h at 200 rpm to ensure complete hydrolysis. The mixture was allowed to cool and the hydrolysate was neutralized with 6 mol L^−1^ HCl. 95% ethanol was added to precipitate hemicelluloses and glucomannans. The precipitate was separated by the process of filtration and excess ethanol was removed with the help of a rotary vacuum evaporator. A brown‐colored extract containing FA was obtained and filtered using a 0.45 μm filter. After that, HPLC was used to determine the quantity of FA in the extract. It was observed that the extraction efficiency of FA increased with time and increasing temperature, but decreased with increasing pH.^18^, ^19^

### Estimation of FA

#### Spectrophotometric technique

Spectrophotometry is the quantitative assessment of the interaction of ultraviolet, visible and infrared light with material, with numerous applications in technology and science. The nature of this contact is determined by the material's physical qualities, such as whether it is transparent or opaque, smooth or rough, pure or polluted, thin or thick. Thus, spectrophotometric data may be utilized to characterize the material's key physical characteristics.^20^ For quantitative quantification of FA, a simple, sensitive and repeatable spectrophotometric technique based on Folin–Ciocâlteu reagent in 15% sodium carbonate has been established. The blue chromogen generated as a result of the reaction was measured at the wavelength of maximum absorption for FA, at 718 nm, against the blank reagent. Over the range of 1–8 μg mL^−1^, the chromogen followed linearity.^21^ The surface‐enhanced and normal Raman spectra range of FA were 485–1605 a.u. and 578–1629 a.u., respectively.^22^ The ultraviolet absorbance range of most of the phenolic acid was 280 nm whereas FA showed strong absorbance at 320 nm. The *R* ^2^ calibration value and *R* ^2^ cross‐validation were 0.82 and 0.78, respectively.^23^ Spectrophotometric readings of samples ranging from 1 to 8 μg mL^−1^ were taken, with correlation coefficient value of *R* ^2^ = 0.988 and rectilinear regression equation *y* = 0.094*x* − 0.001. The results found for different samples were calculated using a standard curve and the results were as follows: for bamboo shoots, the concentration of FA was 1.7 μg mL^−1^; for wheat bran, the concentration of FA was 7.3 μg mL^−1^; rice bran had the highest concentration of FA, at 8 μg mL^−1^.^24^

#### High‐performance liquid chromatography

HPLC is a form of column chromatography in which a sample mixture or analyte in a solvent (known as the mobile phase) is pushed at high pressure through a column with chromatographic packing material (stationary phase). The sample is transported by a moving carrier gas stream of helium or nitrogen. At trace amounts as low as parts per trillion, HPLC can separate and identify compounds in any item that can be dissolved in a liquid.^25^ FA was estimated using an HPLC technique. The separation was carried out on a column using an acetonitrile–10% acetic acid mobile phase (20:80, v/v). In the range of 200–7000 ng mL^−1^, linear regression analysis revealed a good correlation between peak area and concentration, with a correlation coefficient of *R* ^2^ = 0.996.^26^ FA showed different peak values for different samples in HPLC analysis; for example, in bamboo shoot, wheat bran and rice bran the values were 6.794, 6.804 and 6.791, respectively.^24^ For reverse‐phase HPLC of FA in sugar beet pulp with citrate buffer as eluent (0.01 mol L^−1^, pH 5.4) and methanol (88:12, v/v), the value of the regression equation is close to 1 and the correlation coefficient was >0.9.^27^

## STRUCTURE

**Figure 1:**
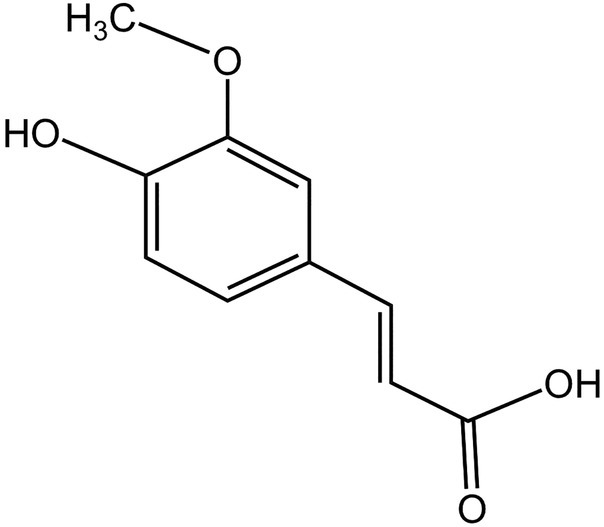
Structure of ferulic acid (Chemspace ID: CSSB00016273339).

FA is an organic compound that is also known by several other names, such as 3‐methoxy‐4‐hydroxycinnamic acid (hydroxyl‐cinnamic acid), caffeic acid 3‐methyl ether and coniferic acid.^28^ The molecular weight of FA is 194.18 g mol^−1^, but it has two other masses – an exact and monoisotopic mass – the value for both being 194.05790880. The compound shows a canonicalization property and its complexity is 224. It contains two hydrogen donor bonds and four hydrogen acceptor bonds; its rotatable bond count is 3, as shown in Fig. 1. However, the structure of FA contains 14 heavy atoms and its topological polar surface area is 66.8 Å.^2^ FA is soluble in water, ethanol, methanol, chloroform, dichloromethane, methyl acetate, ethyl acetate and butyl acetate at a temperature range of 273.15– 333.15 K.^29^

It is one of the most frequent phenolic acids found in plants.^30^ Phenolic compounds are a diverse range of natural chemicals that have an aromatic ring connected to one or more hydroxyl groups. Simple phenols, phenolic acids and polyphenols are the three types of phenolic acid, based on their structures and metabolic processes.^31^ Isoferulic acid and 3‐hydroxy‐4‐methoxycinnamic acid are also produced by plants. Phenolic acids are a type of secondary metabolite with a diverse chemical and biological profile. The esters, lignin components, glycosides and hydrolyzed tannins forms are abundantly found in plants.^32^ It can be separated into cinnamic acid and benzoic acid derivatives with various numbers and substitutions of hydroxyl and methoxy groups, and phenolic acid with peculiar properties in terms of chemical structure. It is the most frequent cinnamic acid derivative, along with caffeic, *p*‐coumaric, syryte, synapine and vanillin acids.^22^

Ferulic acid and its dimer are found in almost every primary plant cell wall. The monomer is covalently linked with monosaccharides and disaccharides, lignin, polyamines, glycoproteins and hydroxyl fatty acids in suberin and cutin. It was first isolated from *Ferula foetida* (Apiaceae) in the mid‐19th century.^22^ In 1866, FA was extracted from a commercial resin for the first time, and it was chemically synthesized in 1925. According to Japanese researchers in the 1970s, FA was isolated from rice oil and showed higher antioxidant activity and several biological benefits such as anti‐aging, anti‐inflammatory, antidiabetic, and many more.^33^ Fundamental constituents such as monosaccharide, disaccharides, glycoproteins, lignin, polyamines, hydroxylated fatty acids and plant cell wall polysaccharides may also be found in the cell walls of many plants, as well as in their seeds and leaves, in both free and esterified form.^34^ The *trans* isomer of FA predominates in several fruits and vegetables, and contributes approximately 90% of total phenolic acids, whereas the FA monomer is the major phenolic constituent in foods such as carrots, tomatoes, corn and oranges.^35^

FA, as well as its precursors *p*‐hydroxycinnamic acid (*p*‐coumaric acid) and 3,4‐dihydroxycinnamic acid (caffeic acid), are metabolites involved in lignin formation. These molecules are intermediates in the biosynthesis of chlorogenic acid, coniferyl alcohol, curcumin, diferulic acids, *p*‐coumaryl alcohol, sinapic acid, synapyl alcohol and vanillin, and can be generated either from tyrosine or phenylalanine through the shikimate pathway.^36^, ^37^ It has antioxidant capabilities because of its phenolic nucleus, which is linked with the C3 side chain. It also slows down numerous biological activities such as aging and pigmentation, and provides health advantages, particularly against severe human disorders and oxidative stress.^38^ The antioxidant activity of phenolic acids (notably cinnamic acid derivatives) is determined by the number of hydroxyl and methoxy groups that are linked to the phenyl ring.^39^ An amide is a derivative of FA that is produced by its condensation with tyramine can use as a plant stress indicator. It is said to reduce the negative effects of chemotherapy and radiation for carcinomas by strengthening the body's natural immunological defense.^40^

## BIOACTIVITY AND HEALTH BENEFITS OF FA

**Figure 2:**
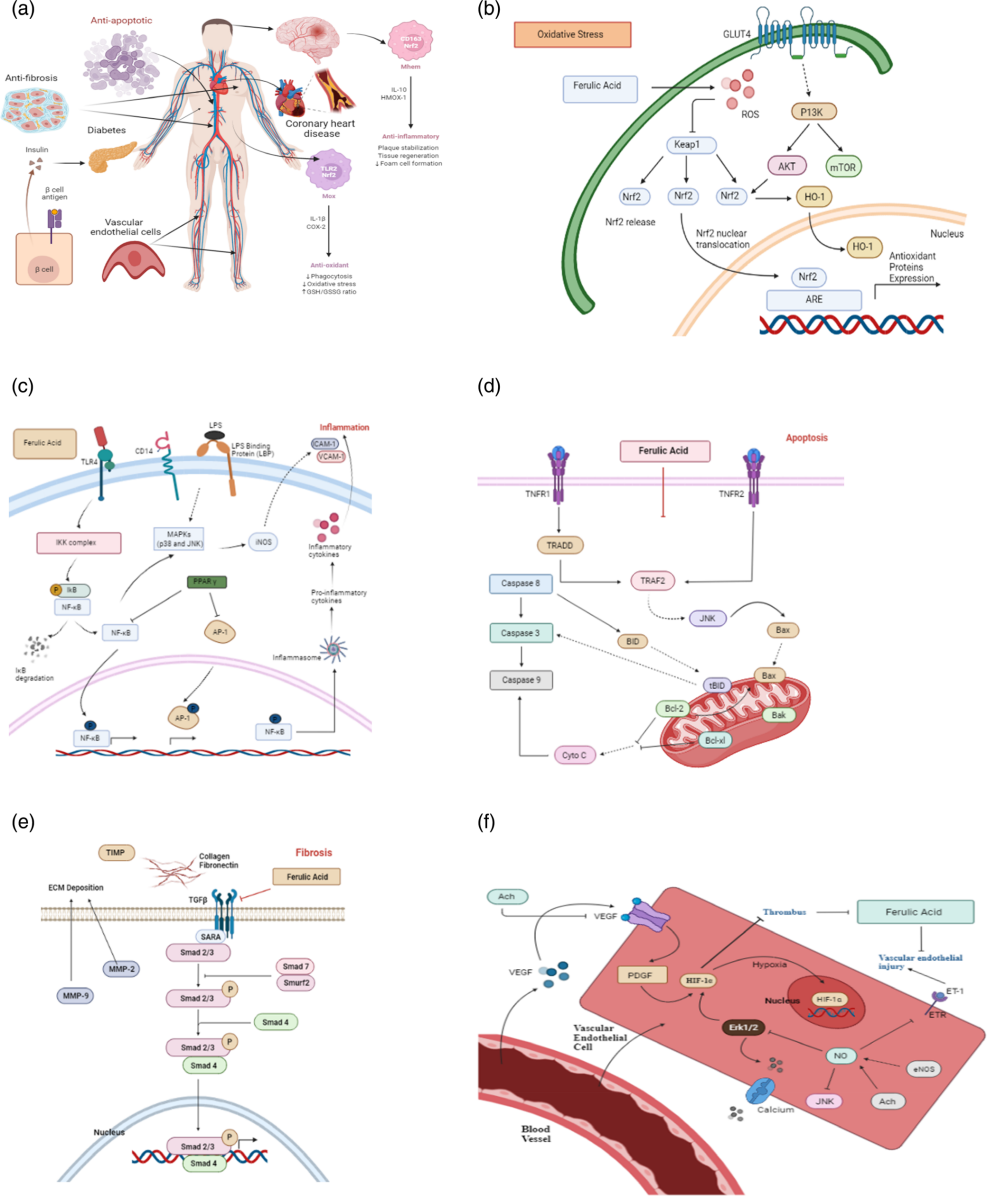
(a) Several health benefits of ferulic acid. (b) Molecular pathway of ferulic acid as antioxidant activity. (c) Molecular pathway for ferulic acid as anti‐inflammatory activity. (d) Molecular pathway for ferulic acid as anti‐apoptotic activity. (e) Molecular pathway for ferulic acid as anti‐fibrosis activity. (f) Molecular pathway involved in ferulic acid protection in vascular endothelial cells.

FA is non‐toxic and performs a variety of physiological activities, including anti‐inflammatory, antibacterial, anticancer (including lung, breast, colon and skin cancer), antidiabetic as well as immunostimulant properties.^41^ It also shows anti‐inflammatory qualities and may aid in the healing of injured nerve cells. FA is also a sports supplement that helps to remove oxidative stress from muscle tissue during fatigue. It is used in the pharmaceutical industry as an antioxidant and anti‐inflammatory agent, and in the food industry for extending the preservation process. It is also used as a photoprotective agent (sunscreen), for slowing the photoaging of skin and as a brightening component in skincare formulations.^42^, ^43^ Figure 2(a) illustrates several health benefits of FA intake.

### Antioxidant

The term ‘antioxidant activity’ has become highly prominent in the modern world and has received much media attention in relation to foods and health benefits.^44^ An antioxidant agent can be defined as ‘any substance or material that opposes oxidation or inhibits reactions driven by oxygen and peroxides, allowing it to be used as a preservative in diverse products’ according to a traditional and broad definition.^7^ FA has been shown to have powerful antioxidant effects. The phenolic ring provides great resonance stability and allows it to receive electrons from free radicals more easily. As a result, it serves as a direct scavenger of free radicals and reduces oxidative stress.^45^ Oxidative stress is the condition of unbalance between oxidation and anti‐oxidation in the body.^46^ It induces neutrophil inflammatory infiltration, and enhances protease release and the generation of a significant number of oxidation intermediates responsible for tissue deterioration, accelerated aging, apoptosis, cell transformation and malignancy.^4^

Nowadays, oxidative stress is assumed to be associated with several diseases related to age, such as cancer, diabetes, hypertension, ischemia–reperfusion damage to various tissues, neurodegenerative diseases, rheumatic diseases and stroke. The antioxidant effect is shown by neutralization of oxidative free radical damage, decrease in reactive oxygen species (ROS) production and interaction in numerous signaling cascades.^3^ The body is in constant touch with oxygen and ROS (an oxygen‐containing compound), whereas the body's anti‐oxidation mechanism can resist oxidation, preventing the accumulation of ROS in the body and help in repairing the harm (excessive ROS can damage cellular proteins, lipids and DNA). When the body's antioxidant mechanism and the creation of ROS become imbalanced, oxidative stress occurs, which leads to the synthesis of a large amount of ROS. In mouse renal podocytes (visceral epithelial cells) generated by high glucose, FA was reported to prevent cell damage via lowering ROS.^47^ Scientists revealed that after 8 weeks of gavage diabetic rats were able to reduce the formation of free radicals, block the growth of intracellular ROS and control variations in oxidative stress index values, i.e. superoxide dismutase (SOD), malondialdehyde and catalase (CAT), in the body.^48^ The production of advanced glycation end‐products (AGEs) and xanthine oxidase activity was reduced by the use of FA; however, it showed an inhibitory effect (both of which are markers of ROS inducers under the pathophysiological situation of diabetes) when compared to untreated diabetic rats. In HEK293 cells exposed to H_2_O_2_, preliminary treatment with FA (1 mmol L^−1^) can considerably reduce ROS levels, boost (CAT) and SOD levels, and improve HEK293 cell viability. As a result, FA helps to control the activity of the antioxidant enzyme and ROS.^3^

In an *in vivo* study, it was observed that a range of cell defense genes were activated and stress initiation was regulated by a redox‐sensitive transcription factor, i.e. nuclear factor‐erythroid‐related factor 2 (Nrf2).^49^ In another report, it was observed that HO^−1^ was degrading carbon monoxide (CO), biliverdin and iron ion (Fe^2+^) from heme, which is a rate‐limiting enzyme in heme catabolism and a crucial substrate for Nrf2 to exercise its antioxidant action.^50^ The Nrf2/HO^−1^ cascade reaction showed a strong inhibitory impact on pathophysiological processes of colon, diabetes, kidney, liver, lung and ischemia–reperfusion damage in animals during oxidative stress,^51^ whereas phosphatidylinositol‐3‐kinase (PI3K) is responsible for generating phosphatidylinositol‐3,4,5‐triphosphate (PIP3), which helps to transport inactivate serine–threonine kinase (Akt) from the cytoplasm to the cell membrane. It binds to PI3K through its pH range,^52^ resulting in 3‐phosphoinositide‐dependent protein kinase‐1 (PDK1), and when active Akt is released into the cell it triggers a signaling cascade that stimulates the downstream target protein mTOR.^3^

Aldose reductase (AR) with nicotinamide adenine dinucleotide phosphate (NADPH) as a cofactor catalyzes the conversion of aldehydes or ketones to matching alcohols. NADPH is linked to oxidative stress and is involved in the onset and progression of diabetes problems as well as a number of oxidative stress illnesses.^53^ When diabetic nephropathy develops, AR activity increases, which causes a high amount of NADPH to be used, harming the body's redox system. FA shows a noncompetitive inhibitory effect (which occurs when an inhibitor attaches to the enzyme somewhere other than the active site) on AR in rat kidney, decreasing AR activity and the formation of AGEs while also lowering transforming growth factor (TGF)‐1 expression and secretion.^3^ The mechanism for the antioxidant role of FA is shown in Fig. 2b.

### Anti‐inflammatory

FA has a powerful anti‐inflammatory impact on a wide range of disorders, such as acute respiratory distress syndrome (ARDS), Alzheimer's disease, endometritis, kidney and liver failure and testicular injury.^54^, ^55^ Severe diseases such as atherosclerosis, cancer, cardiovascular diseases, diabetes and rheumatoid arthritis are thought to be influenced by inflammation and immune responses.^56^ FA primarily controls the production and expression of inflammation‐related factors through numerous biological mechanisms to ameliorate and resist inflammation.^57^, ^58^ It plays a crucial role in the treatment of different immunopathological conditions such as the buildup of pro‐inflammatory cytokines and inflammatory mediators such as ROS and nitric oxide (NO).^57^, ^59^

The nuclear factor kappa‐B (NF‐ κB) is a transcription factor class that controls the transcription of genes involved in cellular proliferation, transformation and death in eukaryotes.^60^ It can lead to inflammation, autoimmune disorders, and possibly cancer and other malignant diseases, if the activity of NF‐κB is not adequately controlled. Depression is also a severe issue that is linked with neuroinflammation.^61^ Scientists revealed that mice experience chronic unpredictable mild stress (CUMS) when microglia are activated, which can enhance interleukin (IL)‐1β, IL‐6 and tumor necrosis factor (TNF) synthesis, resulting in neuro‐inflammation. The immune response was regulated by NF‐κB, which modulates the proinflammatory cytokine signaling cascade (including stress). The inflammatory signal of NF‐κB is activated by CUMS, whereas FA works in the same way as fluoxetine (anti‐inflammatory and pain relief effects). It also acts as an antidepressant, which helps to inhibit the activity of NF‐κB and NLR family pyrin domain‐containing protein‐3 (NLRP3) inflammasomes.^3^

FA therapy shows a similar impact to toll‐like receptor 4 (TLR4) inhibitor (TSK242) and NF‐κB inhibitor (SP600125), which can decrease glial cell excitation and the production of TLR4, Phosphorylated Jun N‐terminal kinase (p‐JNK) and p‐NF‐κB, consequently suppressing the expression of downstream signaling molecules such as inducible nitric oxide synthase (iNOS), cyclooxygenase‐2 (COX‐2), TNF‐α and IL‐1. FA can also block the activation of NF‐κB, whereas COX‐2, iNOS, vascular cell adhesion molecule‐1 and intercellular adhesion molecule‐1 expression was modulated by NF‐κB, suggesting that a supplement that could fight chronic inflammation and aging could be developed.^62^

Lipopolysaccharide (LPS) can activate p38 MAPK, one of the primary signaling mechanisms of mitogen activated protein kinases (MAPKs), which plays a crucial role in inflammatory reactions.^3^ LPS forms a complex compound in the bloodstream when it binds with LPS binding protein (LBP), which then subsequently attaches to the CD14 molecule on the myeloid cell surface. It activates the p38 MAPK, and IL‐1, IL‐6, TNF‐α and other inflammatory cytokine mediators are released by monocyte macrophages and the neutrophils are activated to engage in the inflammation reaction.^63^ It was discovered that FA pretreatment suppressed MAPK activation, whereas LPS is responsible for reducing the mRNA expression of proinflammatory cytokines (IL‐1, IL‐6, TNF‐α and IL‐8), and includes p38 and JNK, in the LPS‐driven inflammatory reaction of bovine endometrial epithelial cells. As a result, in endometritis treatment, FA is used as an anti‐inflammatory medication.^64^

Peroxisome proliferator activated receptors (PPARs) belong to the ligand‐activated nuclear transcription factor superfamily. By blocking the NF‐κB, STAT and AP‐1 pathways, PPAR agonists can reduce the production of inflammatory proteins in monocyte macrophages (including IL‐1, IL‐6, TNF‐α, iNOS and COX‐2).^65^ PPAR is stimulated from primary components such as eicosapentaenoic acid, omega‐3 polyunsaturated fatty acid (ω‐3PUFA) (fish oil) and docosahexaenoic acid. ω‐3PUFA can inhibit LPS‐induced NF‐κB activity and monocyte chemoattractant protein‐1 expression while considerably increasing PPAR mRNA transcription.^3^ Scientists revealed that in methotrexate‐induced nephrotoxicity, FA can upregulate PPAR‐γ and Nrf2 signal transduction and avoid over‐synthesis of ROS.^66^ The anti‐inflammatory mechanism of FA is illustrated in Fig. 2(c).

### Anti‐apoptosis

Apoptosis is a natural approach for eliminating senescent cells *in vivo* as well as a process for maintaining tissue cell balance. Many disorders, including neurological diseases, ischemia injury, autoimmune diseases and cancer, are caused by improper apoptosis (either too much or too little).^67^ According to recent studies, it depends on the two mechanisms: an endogenous mechanism that depends directly on the mitochondria; and an external mechanism that is mediated by the death receptor.^68^ A complete knowledge of the relationship between apoptosis and disease has far‐reaching implications for disease treatment.

Recent research has discovered that FA can act as an anti‐apoptotic role via modulating several targets.^3^, ^54^ By modulating the mitochondrial outer membrane permeability, B‐cell lymphoma‐2 (Bcl‐2) family proteins help to modulate mitochondrial apoptosis, initiating the downstream caspase cascade to accomplish apoptosis.^69^ The two types of anti‐apoptotic proteins that interact and have an impact on human health and illness are anti‐apoptotic proteins (such as Bcl‐2 and Bcl‐XL) and pro‐apoptotic effectors (Bak and Bax).^70^ By enhancing Bcl‐2 expression and lowering Bax expression, FA can improve placental apoptosis. The p53 protein can cause lens opacity by promoting apoptosis in human lens epithelial cells (HLEC). FA (500 mol L^−1^) can prevent HLEC apoptosis by dramatically lowering p53 protein expression and increasing the Bcl–2/Bax ratio.^71^, ^72^ In another study, it is reported that in a model of cerebral artery occlusion FA (100 mg kg^−1^) shows a significant reduction in cytochrome C, caspase‐3, mitochondrial Bax and glial fibrillary acidic protein (GFAP), whereas in cytoplasm and mitochondria the expression ratio of phosphorylated p38 MAPK (p‐p38 MAPK)/p38 MAPK, phosphorylated Bad (p‐Bad) expression and Bcl‐2/Bax was substantially restored.^73^ The mechanism of FA in the anti‐apoptotic role is shown in Fig. 2(d).

### Anti‐fibrosis

Fibrosis is a type of aberrant wound healing induced by organ damage, in which scar tissue forms in the wounded organs and blood vessels. Destruction occurs in the organ structure due to the excess production of extracellular matrix and its deposition in connective tissue and organ tissue, which leads to impairment in its function.^74^, ^75^ The stages of fibrosis can be categorized into four categories: triggered by substantial organ damage, followed by the activation of effector cells, refinement of extracellular matrix and an increase in the growth of fibrosis, and consequently leading to end‐organ dysfunction.^76^, ^77^ It can prevent excessive collagen accumulation, which is a symptom of many fibrosis illnesses. It has been demonstrated to have a substantial anti‐fibrosis impact on liver, lung and renal fibrosis.^78^

TGF‐β is produced and released by effector cells and inflammatory cells, and is an efficient activator of extracellular matrix protein produced in most fibroblast cells. The TGF‐β system, which plays a vital role in fibrosis regulation, can collaborate with the other cell signaling pathways.^79^ A study demonstrated that epithelial–mesenchymal transition (EMT) in pulmonary fibrosis, caused by a single dose of 1.5 mg per animal of crystalline silica (0.5–2 μmol L^−1^), which prevented TGF‐β/Smad signal transduction, could be prevented by a 100–300 mg kg^−1^ dose of FA.^3^ It is observed that EMT of renal tubular epithelial cells is promoted by TGF‐1 in the investigation of renal fibrosis (NRK‐52E). A 25–200 μmol L^−1^ dose of FA can block the expression of the Smad2/Smad3 signal, upregulate E‐cadherin expression and thus hinder the EMT process and prevent fibrosis.^80^ In the fibrosis model, the matrix metalloproteinases (MMPs) or tissue inhibitors of metalloproteinases (TIMPs) system plays a crucial role in the formation and breakdown of ECM. FA can protect the liver from fibrosis caused by alcohol and PUFA via modulating the MMPs/TIMPs system and lowering collagen expression.^81^ By reducing the expression of MMP‐2 and MMP‐9, FA (1 mg per site per mouse) can reduce the breakdown of collagen, aberrant elastic fiber buildup and epidermal hyperplasia (caused by ultraviolet B).^82^ The role of FA in anti‐fibrosis is illustrated in Fig. 2(e).

### Role of FA protection in vascular endothelial cells

The pathophysiology of cardiovascular diseases such as atherosclerosis, coronary heart disease and hypertension is underpinned by pathological vascular remodeling, in which the malfunctioning of vascular endothelial cells (ECs) is the first link.^1^, ^3^ ECs are the basal layer of monolayer cells that come into direct contact with blood constituents and cells. The smooth muscle procreation, tension, coagulation, vascular permeability, release of different growth factors, anticoagulation, immunological reaction and migration are all regulated by ECs, which play a crucial role in blood vessel formation.^83^ According to recent research, ECs are closely linked to vasodilation. Inflammatory stimuli, obesity, oxidative stress and insulin resistance are all responsible for the activation of NF‐κB, whereas raising the expression of iNOS raised the expression and increases the generation of ‘high‐content’ nitric oxide, destroyed vascular endothelial cells and caused vasodilation.^3^ The transduction mechanism is used in the cell signaling of extracellular signal‐regulated kinase 1/2 (ERK1/2) from the surface receptor of the cell membrane to the nucleus. However, it also increases the inflammatory cell proliferation and migration, enhancing the vascular endothelial inflammatory response and resulting in endothelial damage.^84^ Scientists revealed that FA is used to reduce the growth of vascular smooth muscle cells, which is stimulated by angiotensin II via inactivating ERK1/2 and c‐Jun N‐terminal kinase (JNK) (whereas, c‐Jun is the combination of protein). It also inhibits cell proliferation by lowering cyclin D1 expression, whereas it controls the transition of cells from the growth 1 (G1) phase to the synthesis (S) phase.^85^ The mechanical pathway for FA protection in vascular endothelial cells as shown in Fig. 2(f).

### Coronary heart disease

Coronary heart disease is a type of stenosis or blockage of the arterial lumen produced by atherosclerotic lesions of the coronary artery that leads to myocardial ischemia, hypoxia or necrosis. It is also known as coronary atherosclerotic heart disease. The imbalance of vasoconstrictor (endothelin‐1, etc.) and vasodilator (NO, etc.) production and release, produced by endothelial cells, is the key to coronary heart disease.^86^, ^87^ The balance of pro‐apoptotic and anti‐apoptotic proteins is critical to maintain the cardiomyocyte cell state. The Bcl‐2 protein family, caspase family, aspartate‐specific cysteine protease and IL‐1‐converting enzymes all modulate it. By managing relevant elements, FA can effectively maintain body balance and regulate the emergence and progression of illnesses.^88^, ^89^

### Diabetes

Diabetes, characterized by hyperglycemia, is the most common endocrine disorder. Diabetes‐related hyperglycemia can be caused by: (a) reduced glucose entry into cells; (b) decreased glucose utilization by different tissues; and (c) increased glucose synthesis (gluconeogenesis) by the liver.^90^ Hyperglycemia induces an increase in free radical production, which results in oxidative stress. Oxidative stress is an imbalance between quantities of pro‐oxidants and antioxidants in biological systems, which causes cellular damage. FA has been shown to have antioxidant properties, helping to neutralize free radical formation due to hyperglycemia. It is also responsible for the decrease in the level of oxidative stress. This reduction in oxidative stress reduces the breakdown of pancreatic beta cells, improves beta cell function and allows them to release more insulin. This enhanced insulin production can result in higher glucose consumption by extrahepatic tissues, lowering blood glucose levels. FA treatment indirectly helps to lower blood glucose levels.^74^

## FOOD APPLICATIONS

FA has several food applications, including use as a crosslinking agent in food preservation. In the baking sector, amides of FA with amino acids or dipeptides are used as a preservative. The government of many countries (China, Japan and in Europe) have allowed the use of FA as a food additive because it effectively scavenges superoxide anion radicals and reduces lipid peroxidation.^91^ The food sector is under growing customer pressure to avoid the use of synthetic preservatives. The capacity of FA to suppress both fatty acid peroxidation and the growth of bacteria, fungi and yeast suggests that this natural molecule might be employed as a natural food preservative.

It is used as a precursor of vanillin, which is a flavoring agent, and it also provides a good fragrance. It is also used for the preservation of oranges. Industries such as baking, food processing, and chocolate and ice cream manufacture have a high demand for FA. It is frequently utilized as an ergogenic component in sports meals because of its powerful antioxidant activity and its ability to promote hormone release in the human body.^40^

FA has been instrumental in the development of edible films for food and drug packaging, which have gained popularity due to their ability to improve food quality by functioning as aroma, gas, moisture and lipid barriers, as well as protecting food products after the primary packaging is opened. These edible films are biodegradable and can be consumed with food, reducing pollution caused by non‐biodegradable plastic films.^91^ Emerging applications of FA in innovative food technologies include its incorporation into smart packaging systems. These systems can interact with food to indicate spoilage or contamination, thereby extending shelf life and ensuring food safety.^92^, ^93^, ^94^

In the dairy industry, FA is being explored for its potential to enhance the shelf life and nutritional profile of milk and milk products. Moreover, it is being used in the development of functional beverages, offering added health benefits through its bioactive properties.^95^

FA's versatility extends beyond its individual benefits, as it also exhibits synergistic effects when combined with other natural preservatives, such as ascorbic acid and tocopherols, further enhancing its antioxidant capacity and antimicrobial efficacy.^8^, ^96^ This synergy not only boosts the preservation qualities of food products but also aligns with the clean label trend, with consumers preferring minimal and natural ingredients. Additionally, FA contributes to the nutritional value of foods by providing health benefits such as reducing inflammation and potentially lowering the risk of chronic diseases due to its antioxidant properties. As research advances, the potential uses of FA in the food industry continue to grow, promising to meet consumer demands for healthier, safer and more sustainable food products.

## FUTURE PERSPECTIVE

FA is a plentiful substance found naturally in the plant kingdom. It may be generated economically from natural sources. Because of its numerous health advantages, as well as its antioxidant and antibacterial actions, FA's use in food applications is expected to expand in the future. The most recent research updates, as well as future research avenues, are also elucidated in relation to the positive effects of this prevalent phenolic compound for a better understanding of its potential applications in health and disease, which may subsequently aid in the development and design of appropriate dietary recommendations and medications.

## AUTHOR CONTRIBUTIONS

Mukul Kumar: investigation, methodology, formal analysis, writing – original draft, writing – review and editing, visualization. Deepika Kaushik and Ashwani Kumar: investigation, formal analysis, writing – original draft, visualization. Shubham Shubham, Emel Oz, Charles Brennan and Vishal Kumar: writing – review and editing, conceptualization, investigation. Maomao Zeng, Charalampos Proestos, Kenan Çadırcı, Muharrem Bayrak and Sercan Karav: visualization, methodology, formal analysis, investigation. Fatih Oz: supervision, formal analysis, methodology, visualization.

## CONFLICT OF INTEREST

The authors declare that they have no known competing financial interests or personal relationships that could have appeared to influence the work reported in this paper.

## Data Availability

The data that support the findings of this study are available from the corresponding author upon reasonable request.
